# Evolving bioinformatics services – the journey of KPI metrics with Scorpion

**DOI:** 10.1515/jib-2025-0048

**Published:** 2026-05-18

**Authors:** Manuel Feser, Daniel Arend, Sebastian Beier, Marie Bolger, Nils-Christian Lübke, Maria Meister, Lena Steilen, Björn Usadel, Uwe Scholz

**Affiliations:** Leibniz Institute of Plant Genetics and Crop Plant Research, Department Breeding Research, Working Group Bioinformatics and Information Technology, 06466 Stadt Seeland, OT Gatersleben, Germany; Graduate School DILS, Bielefeld Institute for Bioinformatics Infrastructure (BIBI), Faculty of Technology, Bielefeld University, Bielefeld, Germany; Institute of Bio- and Geosciences (IBG-4: Bioinformatics), Cluster of Excellence on Plant Sciences (CEPLAS), Forschungszentrum Jülich GmbH, Wilhelm Johnen Straße, 52428 Jülich, Germany; Bioeconomy Science Center (BioSC), Forschungszentrum Jülich GmbH, Wilhelm Johnen Straße, 52428 Jülich, Germany; Institute of Bio- and Geoscience (IBG-5: Computational Metagenomics), de.NBI & ELIXIR Germany, Forschungszentrum Jülich GmbH, Wilhelm Johnen Straße, 52428 Jülich, Germany; GFBio – German Federation for Biological Data: Bremen, Bremen, Germany; Gesellschaft für wissenschaftliche Datenverarbeitung mbH Göttingen, Göttingen, Germany; Heinrich-Heine-University Düsseldorf, Faculty of Mathematics and Natural Sciences, Institute for Biological Data Science, Cluster of Excellence on Plant Sciences (CEPLAS), 40225 Düsseldorf, Germany

**Keywords:** bioinformatics infrastructure, services, impact evaluation, key performance indicators, monitoring, service infrastructure

## Abstract

Key Performance Indicators (KPIs) are essential for evaluating project success and establishing control mechanisms to monitor development, performance, and user acceptance of services in joint projects. However, the absence of standardized frameworks and effective monitoring tools, combined with service providers’ reluctance due to fears of comparability, has limited their adoption in scientific contexts. To address this gap, we developed Scorpion, a flexible tool for KPI monitoring in project management. Scorpion enables service providers to retain control over their metrics while supporting centralized reporting. It offers both web-based and programmatic access, with features for KPI submission, visualization, and user and service management. Initially created for bioinformatics and biodiversity projects, Scorpion is applicable across diverse domains. It is particularly valuable for initiatives like the German National Research Data Infrastructure (NFDI), where funding agencies require KPI reporting for evaluation. We present the Scorpion framework, highlighting its design principles, features, and potential to improve project management practices. Use cases illustrate how Scorpion enhances KPI monitoring efficiency and accuracy, contributing to better impact evaluation, quality assurance, and informed decision-making in project and service management.

## List of (non-standard) abbreviations

AAI: authentication and authorization infrastructure
API: application programming interface
IAM: identity and access management
KPI: key performance indicator
NFDI: German national research data infrastructure
OIDC: OpenID connect
UI: user interface
REST: representational state transfer
SSO: single sign-on

## 1 Introduction

Scientific infrastructures (digital and analog) are fundamental pillars for innovative research and provide a broad spectrum of services to diverse user communities. Their continued relevance and ability to meet evolving scientific demands hinge upon the sustainable provision of these services, which, in turn, necessitates long-term and reliable funding. However, ensuring the latter strongly depends on the ability to demonstrate the impact and quality of provided services. A central challenge in this context lies in the objective measurement and transparent communication of impact. Key Performance Indicators (KPIs) have emerged also in scientific contexts as a state-of-art means to quantify and showcase service performance and scientific value. Upon the regular analysis of KPIs the project management is able to develop strategic goals for benchmarking and decision-making. KPI monitoring is therefore not only an internal management and feedback method, but also a vital instrument for accountability towards funding agencies and offering an opportunity to uncover hidden potential for development and improvement of services. Yet, the path towards effective and efficient KPI monitoring and reporting is filled with practical challenges, especially in federated scientific infrastructures that include multiple and differently organized service providers. Furthermore, a wide disparity in the levels of professionalization among those service providers is observable. While some providers have adopted mature monitoring solutions and sophisticated data collection workflows, many others still rely on manual processes to aggregate and submit usage metrics. This variation complicates the task for the infrastructure or service coordination teams to orchestrate quality assurance across a diverse service portfolio [1]. Manual KPI submission processes are especially prone to gaps and inconsistencies, arising from common issues such as staff illness, turnover, mistakes in data transfer, or simple oversight. Such deficiencies lead to incomplete or unreliable performance data, undermining both internal quality assurance and the ability to convincingly demonstrate impact to external stakeholders. The situation is further challenged by evolving requirements from funding agencies, who increasingly mandate the provision of KPI measurements as a prerequisite for continued or renewed funding [2]. Consequently, the lack of streamlined, harmonized, and robust KPI collection mechanisms constitutes a critical bottleneck for scientific infrastructures seeking to secure their financial sustainability.

Therefore harmonizing KPIs across a network of service providers and automating submission of KPIs minimizes typical risks and inefficiencies associated with manual processes. Addressing this gap promises significant benefits: it would not only facilitate more accurate and timely performance and impact assessments, but also enhance overall quality assurance within a federated service portfolio managed by a single coordination team. In turn, this would strengthen the case for sustained funding and enable scientific infrastructures to better fulfil their mission in meeting the requirements of stakeholders including funding agencies and scientific user communities.

This paper explores these challenges and presents a potential solution in the form of Scorpion – a tool designed to standardize, automate, and harmonize KPI collection and reporting. KPI harmonization in this context means that all service providers agree on a set of KPIs and report them via their own KPI monitoring. This also involves KPI standardization, whereby best practices emerge among service providers and KPIs are defined in terms of which KPIs are tracked, which method is used to measure KPIs, and on which scale they are measured (e.g. cumulatively or per time interval). We begin by comparing available tools and discussing their respective capabilities and limitations. This is followed by an overview of Scorpion’s architecture, features, and its applications within German national scientific infrastructures. The discussion section evaluates the advantages and potential challenges of Scorpion’s adoption, concluding with an outlook on future developments and the broader implications for the scientific infrastructure providing community.

## 2 Related works

Several tools and platforms exist for monitoring and visualizing KPIs, each with their own strengths and limitations in the context of setting up a federated monitoring. Table 1 provides a summary of the evaluation, while the remainder of this section discusses the compared solutions in detail.

**Table 1:** Comparison of Matomo, Grafana, NocoDB and Scorpion based on various criteria.

	Matomo/Google analytics	Grafana	NocoDB	Scorpion
AAI integration	No	Yes	Only paid tiers	Yes
API access	Yes	Through data sources	Yes	Yes
KPI harmonization	Yes	No	Through use of same templates	Yes
Visualization	Yes	Yes	No	Yes
Collaborative use	Yes	Yes	Yes	Yes

Matomo [3] is a widely used open-source analytics platform that offers several features relevant to the analysis of research services. In terms of authentication and authorization infrastructure (AAI), Matomo primarily supports local user accounts, while integration with OpenID Connect (OIDC), LDAP and SAML is possible only through third-party or paid plugins. For data accessibility, Matomo provides a REST API, enabling retrieval of data for registered services. Regarding KPI harmonization, Matomo allows the explicit specification of key performance indicators, facilitating tailored analytics per service. Its visualization capabilities include a customizable dashboard, supporting diverse presentation needs. Additionally, Matomo encourages collaborative use, allowing multiple users to access the system and enabling multiple applications to report data to the same instance. However, Matomo is limited to tracking web applications, making it unsuitable for aggregating data from heterogeneous sources such as GitHub downloads, citations, or workflow executions. Moreover, Matomo requires individual setup within each tracked application, placing an additional burden on service providers.

Grafana [4] is a widely adopted open-source analytics and monitoring solution, renowned for its rich dashboarding capabilities. It supports multiple authentication mechanisms, including OAuth, LDAP and Basic Authentication, making it adaptable to various environments. It operates exclusively as a dashboard interface and does not serve as a storage backend, but it connects to a range of external service provider databases as data sources. While Grafana excels at visualizing and aggregating data from heterogeneous sources via configurable panels and supports multiple data sources concurrently, it does not provide features for harmonizing KPIs across services. The harmonization and standardization of KPIs must be managed externally, which can pose challenges in federated or collaborative scientific projects. Grafana’s best fit is as a dashboard for a single service provider tracking their own metrics of their own services.

NocoDB [5] is an open-source platform that transforms a Postgres database into smart spreadsheets, facilitating collaborative data management. It was identified within the NFDI as one potential solution to track and aggregate KPIs of a consortium. While NocoDB allows the definition of custom views to restrict and control the information that users can access and edit, its visualization capabilities are primarily limited to spreadsheet-like interfaces. Single sign-on (SSO) functionality is restricted to paid tiers, with the free tier supporting only local user accounts. NocoDB supports multiple users and basic access control but lacks advanced dashboarding, aggregation, or harmonization features for comprehensive KPI monitoring in large, federated projects. NocoDB is a very good fit for a data collection and curation tool for non-technical indicators, such as the number of workshops held or publications produced, where collaborative input and duplication filtering are required.

In contrast to these existing solutions, Scorpion aims to address the specific needs of KPI monitoring of a federated service portfolio as it exists in joint scientific projects. It provides a platform for the harmonization and aggregation of KPIs. Scorpion is designed to support the definition of project-agreed KPIs across diverse data sources and organizational boundaries, enabling consistent monitoring and reporting. By focusing on both KPI harmonization and comprehensive dashboarding within a single system, Scorpion seeks to fill the gaps left by tools such as Grafana and NocoDB, offering a more integrated approach for collaborative scientific environments.

## 3 Application

### 3.1 Service portfolio management in NFDI4Biodiversity

The NFDI4Biodiversity consortium consists of around 50 scientific institutions, museums, natural history societies, state offices, and other institutes and expert groups. It aims to provide a comprehensive service portfolio [1] for mobilizing and handling biodiversity and environmental data. To be successful, the consortium must offer added value to the professional community, specifically by granting access to modern technologies, and tools for archiving, publishing, searching and analyzing data. These services are provided by various organisations within the consortium. As a collective group of service providers they are committed to providing services that meet the community’s requirements and fulfil the consortium’s mission at a certain quality level.

To ensure quality and continuous improvement of this service portfolio, NFDI4Biodiversity has established core processes for the onboarding, monitoring, review and offboarding of services. By doing so, NFDI4Biodiversity adopts in an applicable way best practices for quality and service management from well-known business and industry standards such as ISO 9001 [6] and ITIL [7].

Within these processes, Scorpion is beneficial for the processes of monitoring and reviewing of services. It supports the onboarding phase by collecting key information from providers and enables continuous monitoring during operation through defined KPIs. It also facilitates structured reviews to assess quality and performance. In the offboarding phase, Scorpion helps document the service history and provides transparency for decision-making.

By serving as a central collection point for KPI measurements, Scorpion supports the service providers themselves by providing them with data on the usage and or performance of individual services. This is in particular useful when the service provider is a small institution that does not offer a comparable tool itself. At the consortium level, the collected KPI measurements are used in the review process to identify areas for improvement and define necessary actions. In other words Scorpion facilitates the review process for the service portfolio management within the consortium. This is beneficial for the consortium in two important ways. First, the consortium is able to fulfil KPI reporting requirements for the funding agency in a structured and simplified way. Secondly, the results of the service reviews support the consortium’s decision-making bodies to safeguard the strategic development of the service portfolio.

### 3.2 Automated service monitoring in de.NBI/ELIXIR-DE

The de.NBI/ELIXIR-DE network serves as a key provider of bioinformatics services for the life sciences community across Germany and Europe. To maintain a high-quality, relevant service portfolio, de.NBI implements structured processes for both the selection and deselection of services [8]. The selection process begins by ensuring that each proposed service is affiliated with a de.NBI service center or associated partner, with clearly defined responsibility and a designated contact person. Scientific relevance, methodological soundness, and demonstrable added value for the community are essential criteria. A critical requirement is the regular collection and reporting of Key Performance Indicators (KPIs), which provide objective evidence of service usage, impact, and maturity. Only services that meet these standards and successfully complete a formal evaluation are included in the de.NBI portfolio. Deselection is equally systematic. Services that consistently fail to meet established quality criteria – such as persistent unavailability, outdated content, or lack of KPI monitoring – are flagged as “Orphaned.” After a review period, these services may be removed from the portfolio to ensure that de.NBI continues to offer only reliable, up-to-date, and actively maintained resources.

A possible use case of service monitoring can be shown by the Institute of Bio- and Geosciences (IBG-4: Bioinformatics) at Forschungszentrum Jülich (FZJ) being part of the GCBN service center of de.NBI & ELIXIR Germany. This service provider manages a diverse set of services, including Helixer, MapMan, Mercator4, PlabiPD, and Trimmomatic. The need to consistently report KPIs for these services, which range from web-based applications to standalone software, drove the development of an automation pipeline. This pipeline, encapsulated in a Python script, was designed based on these use cases to handle the entire Extract, Transform, and Load (ETL) process for submitting service metrics to Scorpion. The automation is broken down into four key stages: gathering, transformation, submission, and scheduling. The process starts by collecting raw metrics from multiple sources, each representing different aspects of service performance. For web services like Helixer and PlabiPD, the script retrieves user engagement data from the Matomo API. For standalone software such as Trimmomatic and MapMan, it gathers download counts for all releases from GitHub or Matomo as one KPI. Academic impact is measured by scraping Google Scholar citation counts via SerpApi summarizing multiple publications where necessary. The collected data is then mapped to Scorpion’s standardized KPI names, ensuring consistency across services. After transformation, the script submits the measurements as JSON payloads to Scorpion’s REST API using a pre configured API token. Both test (“dry run”) and live submission modes are supported. The entire ETL workflow is automated with a monthly cron job, ensuring regular, accurate, and timely KPI reporting without manual effort.

This automation eliminates repetitive tasks, minimizing human error and ensuring consistency across reporting cycles. Since metrics such as citations can only be captured as monthly snapshots, the scheduled pipeline guarantees up-to-date, reliable data without manual intervention. This approach enhances transparency and decision-making for service portfolio management. Furthermore, the modular design of the automation pipeline enables other service providers with similar infrastructures to adopt and tailor these components, promoting scalability, standardization, and best practices across the broader bioinformatics service community.

## 4 Architecture

**Figure 1:**
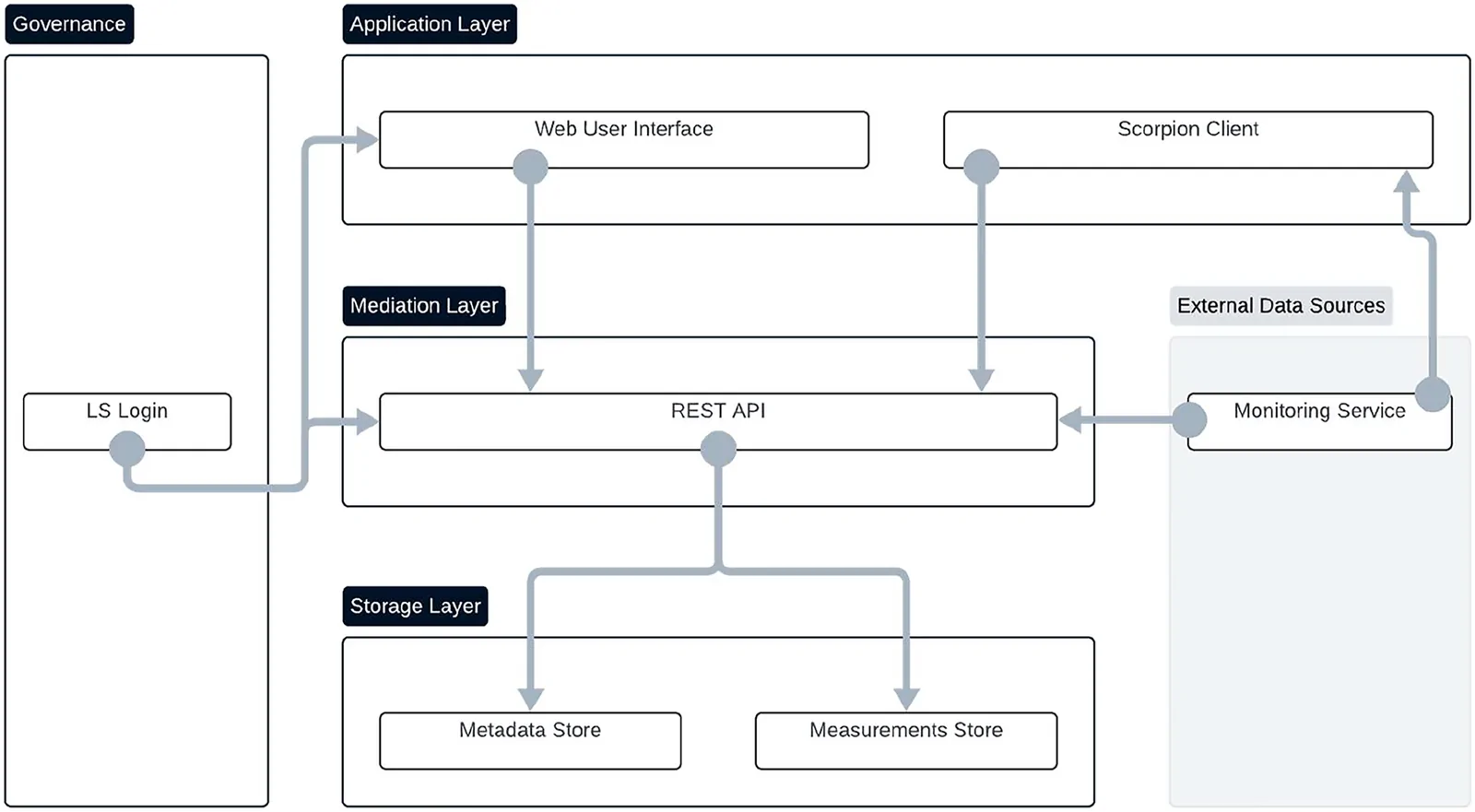
Scorpion can be distinguished into three layers. The application layer provides two services, the Web User Interface and the Scorpion Client, which facilitate direct user interaction. Both of these services communicate with the REST API in the mediation layer, which serves as a central point of contact with the databases. The databases, namely Neo4j and InfluxDB, collectively constitute the storage layer. Identity and access management (IAM) is facilitated through a connection to the LS Login. External monitoring services can submit their measurements either through the Scorpion Client or directly interact with the REST API.

Scorpion uses a layered microservice architecture (see Figure 1) that is designed to support flexible integration and scalable KPI management. Scorpion itself does not track KPIs for services. Instead, service providers register their services within the platform and track the relevant KPIs independently. The providers then push their aggregated measurement data to Scorpion, which acts as a central repository for all KPI-related service information. The system is structured into three distinct layers. The application layer delivers user-facing functionalities. The mediation layer transforms user input into the internal data model to ensure consistency. The storage layer securely persists both metadata and KPI measurements. A governance component manages access control, ensuring that only authorized users can view or enter data. This architecture enables project coordinators to efficiently access and evaluate the collected data, supporting informed decision-making regarding individual services or the broader project.

### 4.1 Application layer

**Figure 2:**
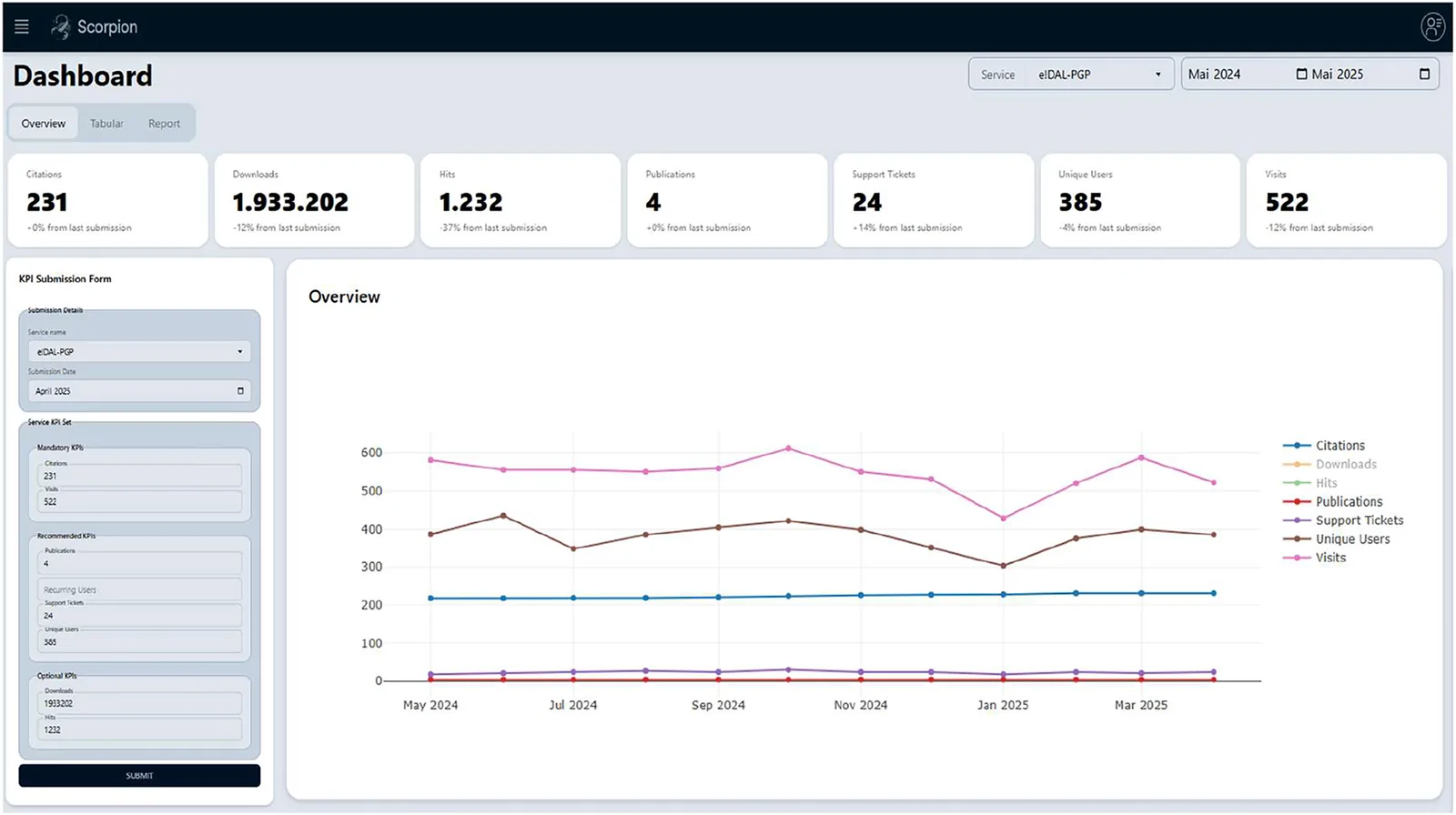
Composed screenshot of the dashboard and the KPI submission form. Users can select the month range to visualize the KPI measurements for a specific service. The dashboard then displays a customizable time plot as well as a tabular view and a report of those measurements. The left side shows the KPI web submission form which shows the different necessities of the KPIs.

The application layer of Scorpion features a comprehensive web user interface (see Figure 2), which serves as the central access point for users. Through this interface, users can register services by providing relevant metadata. This metadata may include the service’s license, development stage, and association with organizational groups, such as NFDI consortia or de.NBI service centers/nodes depending on the configuration of the Scorpion instance. During the registration process, the service provider defines a KPI set for the service by selecting an appropriate service category, which automatically determines the corresponding set of KPIs. Optionally, providers may supplement this set by adding further indicators from other categories to enable a more detailed assessment of the service’s impact. Once a service is registered, the responsible provider can submit KPI measurements to Scorpion. This can be accomplished through using the KPI web submission form, where the provider selects the relevant service from a list associated with their affiliations. The form then displays the service KPI set, allowing the service provider to submit measurements for a specific month. Alternatively, providers may submit KPI measurements programmatically via Scorpion’s REST API, which requires an API token, which can be generated through the account management page. In addition to token generation, users can here request membership to specific service providers – a prerequisite for submitting KPIs for those services. A dedicated administration section allows instance administrators to approve or reject membership requests. Administrators also have access to advanced evaluation and assessment tools, for example for analyzing organizational groups within the infrastructure and the associated service metadata. The Scorpion submission tool is a Python-based command-line utility that allows automated submission of KPIs from Matomo [3] and facilitates citation scraping for scientific publications using SerpApi [9].

### 4.2 Mediation layer

The mediation layer acts as middleware between user-facing services and storage components. It provides a REST API that enforces system consistency by verifying which services are authorized to store specific KPIs. The mediation layer manages the proper persistence of incoming data by distinguishing between metadata and time series measurements. The layer handles data transformation to translate user input and service submissions into the appropriate formats for the underlying storage components, which guarantees data integrity and seamless interaction throughout Scorpion’s architecture.

### 4.3 Storage layer

The storage layer of Scorpion consists of two specialized databases that manage different data types. A Neo4j database stores metadata, including user memberships within service providers, relationships between service providers and consortia, service categories, associated KPI sets and additional KPIs for each service. Using Neo4j allowed to overcome challenges posed by traditional RDBMS solutions such as many to many relationships or changing data models and to implement the tool alongside changing requirements by stakeholders. As time series data is not well suited for a graph database, an instance of InfluxDB is used to record KPI measurements submitted by service providers for their respective services. Using InfluxDB allowed the efficient storage and retrieval of measurement data as well as the clean separation of the metadata and data within Scorpion. By following this dual-database approach, complex metadata relationships and KPI time series data is handled efficiently, supporting the platform’s scalability and data integrity requirements.

### 4.4 Governance component

The governance component of Scorpion ensures robust access control and authorization management. General user authentication is handled via an AAI connected using OIDC; LifeScience Login [10] serves as the AAI provider. This setup enables centralized access authorization through LifeScience Login. Specific feature-level permissions are managed internally by Scorpion, which allows for flexibility in case the AAI provider changes in the future or projects want to connect their own AAI provider. Additionally, API tokens are supported to facilitate secure and streamlined REST API usage. This dual approach guarantees secure access control over system features.

## 5 Discussion

Impact evaluation and KPI monitoring have become central to the management and governance of scientific infrastructure projects. Funding agencies increasingly require regular, standardized reporting to ensure continued support, thereby emphasizing accountability and transparency. However, many scientific infrastructures lack efficient systems for capturing and reporting relevant KPIs. This hinders objective assessments, benchmarking, and strategic decision-making. Robust KPI monitoring demonstrates the value and effectiveness of services, guides resource allocation and improvement efforts, and is an essential component of project management.

Scorpion is designed to streamline KPI monitoring for both service providers and governance bodies. With both programmatic API and a user-friendly graphical interface, Scorpion can accommodate a wide range of workflows and supports automated as well as manual KPI submissions. It provides tools to connect to established solutions such as Matomo and allows for the development of custom scripts that enable seamless integration. The centralization and harmonization of data interfaces enhance efficiency and impact assessment. Importantly, Scorpion’s framework ensures that providers maintain control over their data, addressing privacy and autonomy requirements and concerns. This combination of flexibility, interoperability, and provider-centric control establishes Scorpion as a robust KPI management tool.

An effective evaluation of KPIs in scientific services depends fundamentally on establishing a harmonized set of indicators through stakeholder consensus. The de.NBI project exemplifies this process. After extensive discussions among partners, the project developed a set of KPIs that was agreed upon by all parties [8]. This common/shared set of KPIs ensures comparability, transparency, and meaningful benchmarking across diverse services. Looking forward, achieving broader – potentially international – consensus on harmonized metrics would be a significant milestone. Especially as several indicator lists are defined within different infrastructures [8], 11], 12] and alignment is crucial to avoid divergence. This would facilitate cross-infrastructure participation in KPI monitoring programmes and promote best practices in service assessment. Yet, reaching an agreement will require ongoing dialogue and collaboration among all stakeholders to balance generalizability with the specific needs of individual service contexts and to reduce concerns regarding comparison to and evaluation against other services.

The assessment of KPIs in scientific services presents several challenges that limit the effectiveness of simple, snapshot evaluations. Taking Goodhart’s Law into account, that “an observed statistical regulatory will tend to collapse once pressure is placed upon it for control purposes” [13], 14], reporting single, absolute KPI values to report a service’s impact renders it ineffective as service providers will start to optimize the KPI value to reach the highest possible value. Besides, the reported absolute KPI values can be misleading because they are heavily influenced by differences in user community size and service context, which makes direct comparisons across services undesirable. Therefore, meaningful impact assessment requires long-term analysis to track changes and trends over time. This allows a better understanding of each service’s development by taking into account its life cycle stage and other contexts. Focusing on temporal trends rather than static values allows for more accurate assessment, informed decision-making, and user/community targeted improvements within the scientific service landscape.

Scorpion is currently in an early phase of development, and the first finalized version is now available. Currently, registering new services requires manual input. Although a REST API has been implemented to support programmatic service creation, integration with existing registries and automated workflows are still ongoing. These enhancements are expected to streamline the onboarding process, reduce administrative effort, and improve the overall user experience. Continued development and community feedback are essential to addressing current limitations and ensuring that Scorpion meets the evolving needs of its users.

The ongoing development of Scorpion focuses on expanding its functionality to better support service reviews and evaluations. Planned features include advanced visualization tools to facilitate more insightful analysis during the review process, automated evaluation report generation, and customizable report cards for reviewers. Integration with service registries, such as plug-ins that enable direct service creation, will further streamline workflows and reduce administrative overhead.
